# The Role of Alpha Cells in the Self-Assembly of Bioengineered Islets

**DOI:** 10.1089/ten.tea.2020.0080

**Published:** 2021-08-16

**Authors:** Fredrik C. Wieland, Mireille M.J.P.E. Sthijns, Thomas Geuens, Clemens A. van Blitterswijk, Vanessa L.S. LaPointe

**Affiliations:** MERLN Institute for Technology-Inspired Regenerative Medicine, Maastricht University, Maastricht, Netherlands.

**Keywords:** alpha TC1-clone 6, HUVECs, INS1E, pseudoislets, self-assembly

## Abstract

Vascularization is undoubtedly one of the greatest challenges in tissue engineering. Its importance is particularly evident when considering the transplantation of (bioengineered) pancreatic islets of Langerhans, which are highly sensitive to the delivery of oxygen and nutrients for their survival and function. Here we studied pseudoislets of Langerhans, which are three-dimensional spheroids composed of β (INS1E), α (alpha TC-1), and endothelial (HUVEC) cells, and were interested in how the location and prevalence of the different cell types affected the presence of endothelial cells in the pseudoislet. We hypothesized that alpha (α) cells play an essential role in islet self-assembly and the incorporation of endothelial cells into the pseudoislet, and are thus important to consider in tissue engineering or regenerative medicine strategies, which typically focuses on the insulin-producing beta (β) cells alone. We first determined the effect of changing the relative ratios of the cells and found the cell distribution converged on a steady state of ∼21% α cells, 74% β cells, and 5% endothelial cells after 10 days of culture regardless of their respective ratios at seeding. We also found that the incorporation of endothelial cells was related to the pseudoislet size, with more endothelial cells found in the core of larger pseudoislets following a concomitant increase of α cells and a decrease in β cells. Finally, we observed that both endothelial and β cells were found adjacent to α cells significantly more frequently than to each other. In conclusion, this study demonstrates that the self-assembly of a pseudoislet is an intrinsically cell-regulated process. The endothelial cells had preferential proximity to the α cells, and this persisted even when challenged with changing the cell ratios and numbers. This study gives insight into the rules governing the self-organization of pseudoislets and suggests an important role for α cells to promote the incorporation of endothelial cells.

**Impact statement:**

Pancreatic pseudoislets, which are three-dimensional spheroids composed of alpha, beta, and endothelial cells, can be formed in microwells and used as a model system to study how the different cell types organize and interact. In this study, we showed that including the glucagon-producing alpha cells (and not only the insulin-producing beta cells) could positively influence the number of endothelial cells in the pseudoislet. This is important for tissue engineers who aim to generate a *de novo* cell source of bioengineered islets for transplantation.

## Introduction

Diabetes mellitus type 1 is an autoimmune disease that results in the depletion of the pancreatic beta (β) cells that produce insulin to regulate glucose uptake. Current therapies include an islet transplantation, but considerable cell loss is observed post-transplantation and there is a shortage of donors. Future (tissue engineered) therapies are based on newly generated cell sources. For example, pancreatic endocrine cells can be differentiated from pluripotent stem cells and aggregated with supporting cells.^1–4^ It has been shown that incorporating endothelial cells in bioengineered islets improves their function upon implantation.^2,5–7^ We were therefore interested to better understand how endothelial cells interact with the two major cell types of the pancreatic islet, namely the alpha (α) and the β cells.

Endothelial cells have multiple important roles in the pancreatic endocrine tissue. They are involved in the establishment of the islets of Langerhans' vasculature and abnormalities in the endothelial network are associated with changes in islet physiopathology.^8,9^

Beyond vasculature, endothelial cells also provide molecules that are involved in proliferation and differentiation during pancreatic development.^10,11^ For example, they produce laminin-1 and instructive signals to initiate pancreatic and duodenal homeobox 1 (Pdx1)-producing cells to differentiate into insulin-producing endocrine cells.^12–14^ In general, endothelial cells are known to improve the function of endocrine cells, for example, by providing extracellular components such as type IV collagen, laminins, and connective tissue growth factor that have been shown to improve insulin secretion and β cell differentiation.^11,15,16^

The interaction between endothelial and β cells, the most prevalent cell type within islets, has been well studied. On a functional level, β cells cocultured with endothelial cells have a sixfold higher glucose-stimulated insulin secretion (GSIS), compared with β cells alone.^17^

These cell types are known to signal to each other within islets. For example, β cells produce factors such as vascular endothelial growth factor (VEGF) to attract endothelial cells to promote islet vascularization. *In vivo* experiments in which VEGF expression was knocked down in β cells resulted in the loss of endothelial cells in the endocrine tissue. Although the β cells remained functional, their depleted VEGF expression resulted in GSIS impairment in the islets.^18–20^ This can be because of the fact that the depletion of endothelial cells in the islet caused a loss of the basement membrane, the components of which (e.g., laminin-411 and -511) have been shown to increase insulin secretion.^20,21^

In contrast, very little is known about the interaction between endothelial cells and α cells, the second most abundant cell type in islets. This establishes the need to better understand the role of α and β cells in the incorporation of endothelial cells into the islet.

In addition to endothelial cells, α and β cells can organize into specific (sub-) structures of the islet, which has been shown to impact its function, mainly insulin secretion.^22^ Rodent islets have an established core of β cells and mantle of α cells, unlike human islets, which are described as having a more random arrangement of endocrine cells,^23,24^ although this has been challenged.^22,24^ Alternatively, there are reports that human α and β cells are organized in small clusters separated by vascularized connective tissue^25,26^ or that the endocrine cells are organized in thin cell layers instead of clusters with a core-mantle arrangement like rodent islets, but where the α cells are arranged along the capillary network.^22^ What is certain is that when the normal organization is perturbed, there is diminished insulin secretion in both murine and human models.^27–29^

To understand the role of α and β cells in the incorporation of endothelial cells in pseudoislets, we investigated their respective spatial arrangement and observed whether specific arrangements or affinities affected the incorporation or localization of the endothelial cell population. With this in mind, we challenged the pseudoislets by tuning the initial seeding population with different ratios of α, β, and endothelial cells, and by changing the size of the pseudoislet. Altogether, this information can lead to a better understanding of how the α and β cell ratio and the inclusion of endothelial cells can support future bioengineered islets.

## Research Design and Methods

### Cell culture

Alpha TC1 clone 6 (ATCC Cat. No. CRL-2934, RRID:CVCL_B036), referred to as α cells (ATCC, Manassas, VA), were cultured in DMEM 6046 (Sigma-Aldrich) supplemented with 10% (v/v) fetal bovine serum (FBS; Sigma-Aldrich), 15 mM HEPES, 0.1 mM nonessential amino acids (NEAA), 1.5% (wt/vol) sodium bicarbonate, and 2.0% (wt/vol) glucose. INS-1E (RRID:CVCL_0351), referred to as β cells (AddexBio, San Diego, CA), were cultured in RPMI-1640 (Gibco) supplemented with 5% (vol/vol) FBS, 1 mM sodium pyruvate, 10 mM HEPES, and 0.05 mM 2-mercaptoethanol. Human umbilical vein endothelial cells (HUVECs) (C2519A, used at passage 5; Lonza, Walkersville, MD), were cultured in EGM-2 (PromoCell; C-22111). All cell types were negative for mycoplasma contamination (Mycoplasma Detection Kit, #B39035; BioTool) and were cultured under a humidified atmosphere with 5% CO_2_ at 37°C.

### Pseudoislet formation

To form pseudoislets, which are three-dimensional aggregates of α, β, and endothelial cells, a microwell array was formed from agarose within the wells of a 24-well plate to create 450 microwells per well, as previously described.^30^ Each well was washed twice with a modified EGM-2 medium supplemented with a final concentration of 10 mM HEPES, 1 mM sodium pyruvate, 0.1 mM NEAA, 2% (wt/vol) glucose, and 0.05 mM 2-mercaptoethanol before seeding the cells. Cell number was determined by trypan blue exclusion on an automated cell counter (TC20; Bio-Rad Laboratories). All cell types were seeded simultaneously into the microwell array, which was then centrifuged at 200 *g* for 4 min to evenly distribute the seeded cells into the microwells. The pseudoislets were cultured up to 10 days in modified EGM-2 medium, with half of the medium volume refreshed daily.

### Immunohistochemistry

Pseudoislets were fixed in 4% (wt/vol) formaldehyde diluted in PBS for 30 min at room temperature (RT), washed twice with PBS, and flushed out of the microwell array into microcentrifuge tubes. The pseudoislets were permeabilized with 3% (vol/vol) Triton X-100 at RT for 60 min, washed twice in washing solution containing 1% (wt/vol) BSA and 1% (vol/vol) Tween 20 diluted in PBS, and blocked in 5% (vol/vol) donkey serum diluted in PBS at RT for 60 min. Primary and secondary antibodies (Supplementary Table S1; all antibodies were validated on both positive and negative controls) were diluted in the washing solution and incubated overnight at 4°C. The pseudoislets were transferred to CELLview dishes (Greiner Bio-One) and mounted in ProLong Gold antifade mounting medium (Invitrogen).

### Spatial arrangement analysis

Optical sections (z-stacks) of the pseudoislets were obtained on a Nikon Eclipse Ti-E inverted microscope equipped with a 40 × /1.3 NA immersive oil objective (Nikon Instruments) and spinning disc X-Light2 (CrestOptics). We analyzed 50 μm in depth of the pseudoislets. Quantification was performed on digital images using Fiji software,^31^ and the optical slice was manually analyzed by using Fiji plugin “cell counter”^31^ (Supplementary Figs. S1 and S2). The quantified cell counts were normalized using a convex combination to calculate a weighted mean.

### Proliferation assay

To label proliferating cells, the ClickIT plus EdU cell proliferation kit Alexa Fluor 647 (Invitrogen) was used according to the manufacturer's protocol. The cells were cultured with EdU for 48 h before the endpoint (either day 5 or 10), after which the pseudoislets were fixed in 4% (wt/vol) formaldehyde diluted in PBS for 30 min at RT, washed twice with PBS, and flushed out of the microwell array into microcentrifuge tubes. Pseudoislets were taken up in 25 μL of FBS, and pipetted into a cryomold containing optimal cutting temperature compound (OCT), and were frozen in liquid nitrogen. Twenty micrometer sections were cut on an adhesive film using a modified Kawamoto method^32^ to help preserve the structure of the pseudoislets during sectioning. Sections were stained with DAPI and mounted with ProLong Gold antifade mounting medium.

### Cell viability assay

Pseudoislets were cultured for 10 days. LIVE/DEAD fixable yellow dead cell stain kit (Invitrogen) was used according to manufacturer's instructions. Pseudoislets were fixed in 4% (wt/vol) formaldehyde diluted in PBS for 30 min at RT, washed twice with PBS, and flushed out of the microwell array into microcentrifuge tubes. Pseudoislets were taken up in 25 μL of FBS, and pipetted into a cryomold containing OCT, and were frozen in liquid nitrogen. Twenty micrometer sections were cut on an adhesive film using a modified Kawamoto method,^32^ to help preserve the structure of the pseudoislets during sectioning. Sections were stained with SYTOX orange nucleic acid stain (Invitrogen) and mounted with ProLong Gold antifade mounting medium.

### Statistical analysis

Data were reported as mean ± SEM or 10th–90th percentile from three independent experiments, each of which included a set of 13 pseudoislets. Statistical analysis was carried out in Prism software (version 8.1; GraphPad, La Jolla, CA), with significance determined by an unpaired Welch's and Holm–Sidak *t*-test when *p* ≤ 0.05.

## Results

### Cell distribution and localization in pseudoislets

**FIG. 1.**
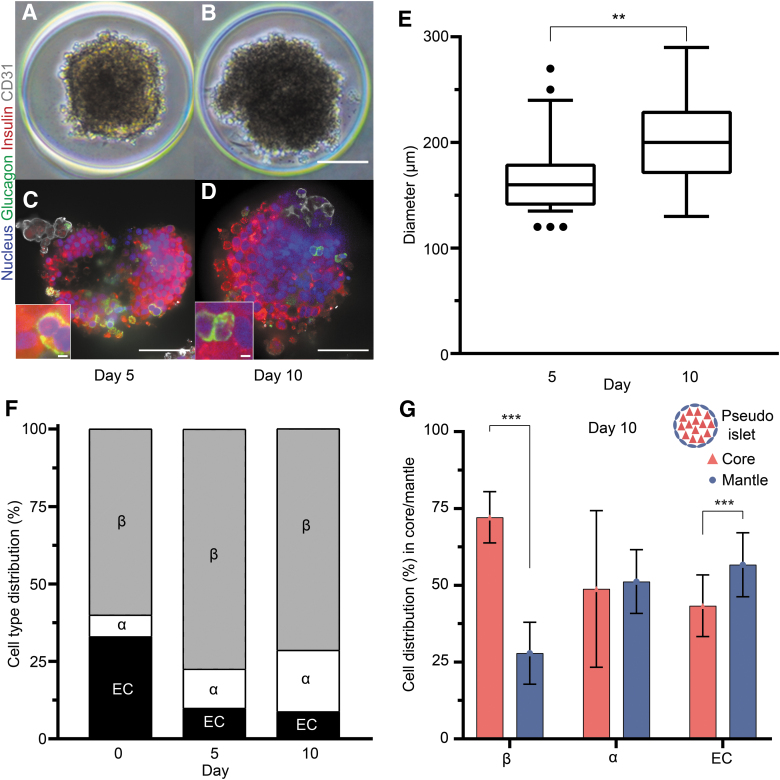
Formation of pseudoislets and their cell type distribution and localization after culture. **(A, B)** Brightfield images of pseudoislets formed by the aggregation of 7% α cells, 60% β cells, and 33% endothelial cells are shown on day 5 **(A)** and day 10 **(B)**. Immunohistochemistry staining **(C, D)** revealed the prevalence and distribution of the different cell types inside the pseudoislet at day 5 **(C)** and day 10 **(D)**, showing nuclei (DAPI; *blue*), α cells (glucagon; *green*), β cells (insulin; *red*), and endothelial cells (CD31; *gray*). Scale bars: 100 μm. Additional zoomed panels show what is considered an interaction in our study. Scale bars: 3 μm. **(E)** The mean diameter of the pseudoislets significantly increased from 173.5 ± 8.0 to 203.3 ± 6.1 μm, during culture (**p < 0.004). **(F)** The cell distribution in the pseudoislet, given as percentages relative to total cells, changed from seeding (day 0) to day 10 in culture. The prevalence of α cells increased steadily over time (additional information in Supplementary Fig 2A, B). Both β and endothelial cells showed significant changes between days 0 and 5, which persisted at day 10. **(G)** The β cells were predominantly localized in the core of the pseudoislets, whereas endothelial cells were localized in the mantle after 10 days in culture (***p < 0.001). The α cells were evenly distributed across the core and mantle. The illustration above the key shows how the core and mantle are defined in the pseudoislet. Results are expressed as mean ± SEM or 10–90 percentile and each data set includes 13 pseudoislets (*n* = 13), and the experiment was repeated three times (*N* = 3).

We created pseudoislets to study the role of α and β cells with regard to the endothelial cells and how their contributions affect the pseudoislet structure. To create the pseudoislet, we began by concurrently seeding α, β, and endothelial cells in a ratio of 7%, 60%, and 33% (totaling 1500 cells per pseudoislet) into a microwell plate. After 5 days in culture, all three cell types aggregated into a single pseudoislet (Fig. 1A, C). From 5 to 10 days in culture (Fig. 1A–D), we observed a statistically significant increase in the mean diameter of the pseudoislet, from 173.5 ± 8.0 to 203.3 ± 6.1 μm (Fig. 1E). At day 5, 58% ± 6.2% of cells in the pseudoislets were proliferating and this was reduced to 45% ± 10.6% at day 10 (*p* < 0.002; Supplementary Fig. S5A, B).

To determine whether the ratios of α, β, and endothelial cells changed during pseudoislet formation and culture, we quantified their relative populations using immunofluorescence imaging. From seeding (day 0) to pseudoislet formation (day 5), the percentages of both α and β cells increased, whereas endothelial cells decreased (Fig. 1F; *p* < 0.001). From day 5 to 10, we observed an increase of α cells from 12.6% to 19.8% ± 2.3% (*p* < 0.0016), whereas the ratios of β and endothelial cells remained similar (Fig. 1F and Supplementary Fig. S3A, B).

To determine whether the spatial arrangement of α, β, and endothelial cells changed during culture, we quantified their location as belonging to either the mantle or core. The cells forming the exterior layer, 20 μm deep from the surface of the pseudoislet, were regarded as the mantle, whereas those within the mantle were considered as the core.^22^ At day 5, α cells were found statistically more predominantly in the mantle (68.1% ± 2.9%, *p* < 0.001) compared with the core (Supplementary Fig. S3C). Βeta cells were more prevalent inside the core at days 5 and 10, where we found 72.8% ± 1.8% of all quantified β cells (*p* < 0.001).

In comparison, only 30.1% ± 3.9% of endothelial cells were located in the core, and the vast majority (*p* < 0.001) were in the mantle at day 10. As for the α cells at day 10, they were more evenly distributed in the two regions, with 51.2% ± 6.3% of all quantified cells localized in the mantle (Fig. 1G, *p* < 0.28). In summary, the α cells in the pseudoislet changed their preferential localization from the mantle at day 5 to a more evenly core/mantle distribution at day 10, whereas the prevalence of endothelial cells in the mantle increased at day 10 (Fig. 1G and Supplementary Fig. S3C).

We hypothesized that a preferential proximity between the various cell types might be the driving force for their resulting spatial arrangement. To test this hypothesis, we quantified the localization of each neighboring cell in the immunofluorescence images. We aimed to identify possible interactions toward the other cell types and therefore did not included homologous interactions. The quantified localization data were converted to a percentage to indicate the probability of a neighboring cell type. The conversion was made to make the interpretation of the data as intuitive as possible.

**FIG. 2.**
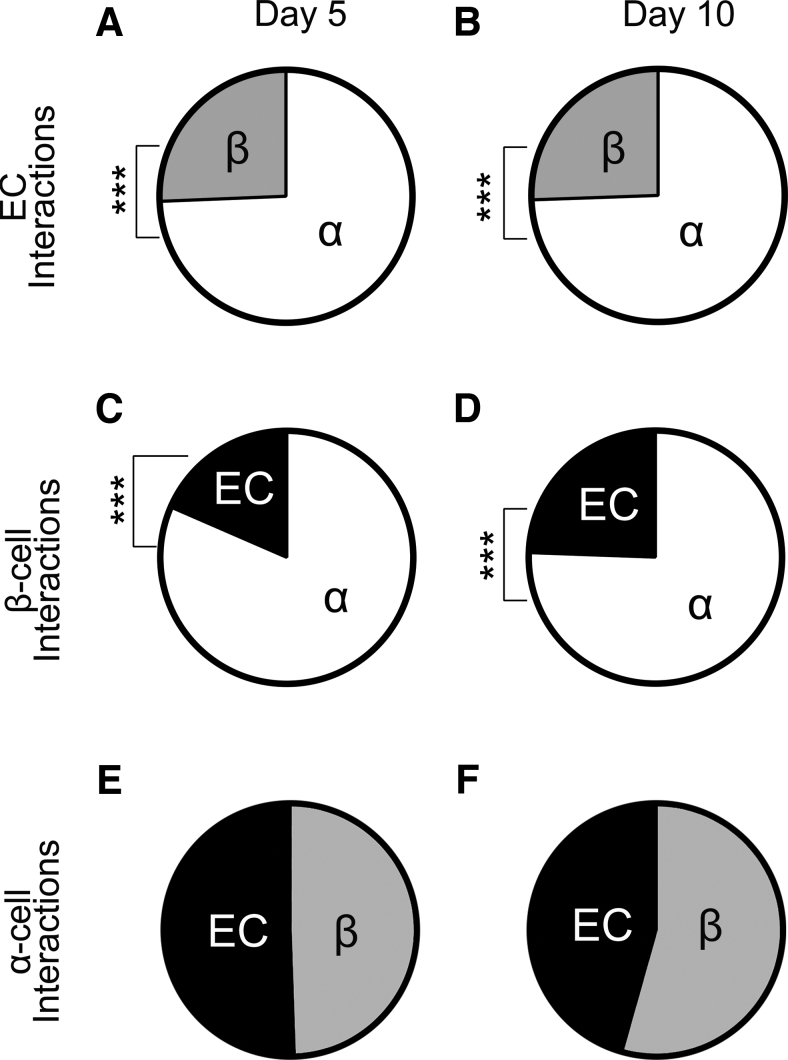
The preferential proximity between the three cell types remained similar from day 5 (*left column*) to day 10 (*right column*) of culture. **(A, B)** Endothelial cells had a preferential proximity to be in contact with α cells although higher numbers of β cells were present (***p < 0.0001). **(C, D)** β cells had preferential affinity for α cells, which did not change during the culture period (***p < 0.0001). **(E, F)** The α cells had an equal preferential proximity to both endothelial and β cells. The localization data were converted to indicate the probability. Each data set includes 13 pseudoislets (*n* = 13), and the experiment was repeated three times (*N* = 3).

Surprisingly, we observed that both β and endothelial cells had a statistically significant preference to neighbor α cells, rather than themselves or each other at both days 5 and 10 (Fig. 2A–D, *p* < 0.0001). This was consistent with their separate localizations, with β cells inside the core and endothelial cells in the mantle. The α cells, on the contrary, were localized between the endothelial and β cells and had an equal affinity to both cell types (Fig. 2E, F). The endothelial cells were three times more likely to be found adjacent to an α cell compared with a β cell, which was especially remarkable given the comparatively low abundance of the endothelial and α cells in the pseudoislet (∼30% combined).

### Pseudoislets attain a cell type equilibrium under different seeding conditions

**FIG. 3.**
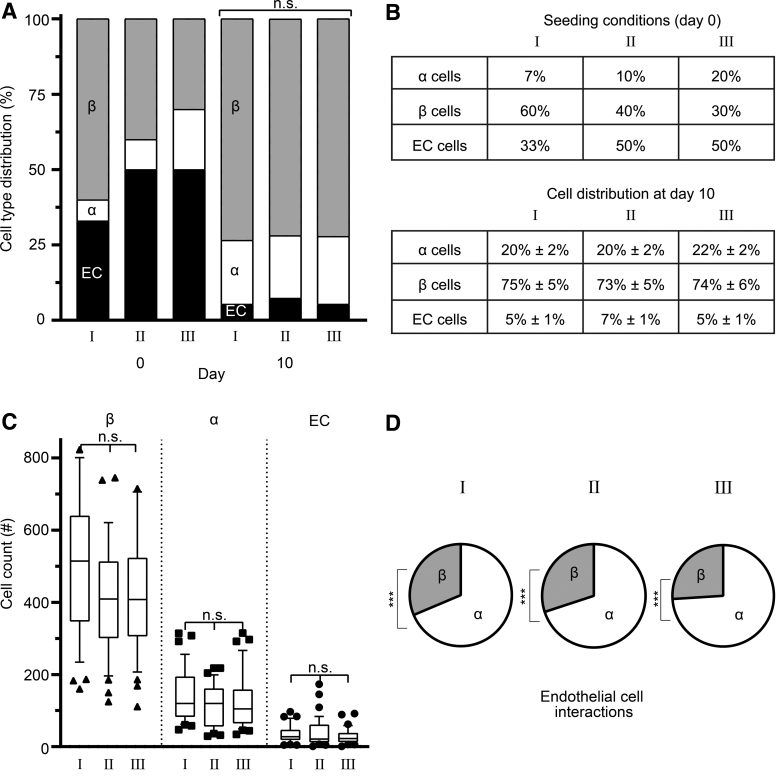
Pseudoislets attained a steady-state distribution of α, β, and endothelial cells. **(A, B)** Three different seeding conditions were used to form pseudoislets. After 10 days, all three conditions converged on a statistically similar cell distribution regardless of the seeding ratios: ∼20% α cells, ∼74% β cells, and ∼6% endothelial cells. **(C)** The three seeding conditions resulted in similar cell counts in the pseudoislets after 10 days in culture. **(D)** The preferential proximity of endothelial cells to α cells was maintained regardless of the seeding density (***p < 0.0001). Results are expressed as mean ± SEM or 10–90 percentile and each data set includes 13 pseudoislets (*n* = 13), and the experiment was repeated three times (*N* = 3). n.s: no statistical significance (*p* > 0.05).

These observations prompted us to concentrate on the preferential proximity between endothelial and α cells. To address this assumption, we first designed multiple conditions to challenge the affinity between the different cell types. We began by altering the composition of the pseudoislet to create three different seeding conditions (Fig. 3B). After 10 days, all three conditions converged on a statistically similar cell distribution, with α cells comprising 20.6% ± 1.8%, β cells comprising 73.9% ± 5.0%, and endothelial cells comprising 5.5% ± 0.7% of the pseudoislet regardless of their respective seeding ratios (Fig. 3A, B). The three different seeding conditions had no effect on the cell counts at day 10, as we found similar counts for all conditions: 127.1 ± 11.1, α cells; 457.5 ± 30.7, β cells; 33.9 ± 4.5, endothelial cells (Fig. 3C). Furthermore, the affinity of endothelial cells to α cells was maintained in all three conditions, with 69–75% of endothelial cells found in proximity to α cells (Fig. 3D and Supplementary Fig. S3D).

### The pseudoislet size influences the prevalence of endothelial cells

To determine whether islet size affects the relative subpopulations of cells, we varied the size of the pseudoislet and determined the α, β, and endothelial cell ratios. We created three differently sized pseudoislets by seeding 750, 1500, or 3000 cells per microwell, all with the composition of 6.7% α cells, 60% β cells, and 33.3% endothelial cells.

**FIG. 4.**
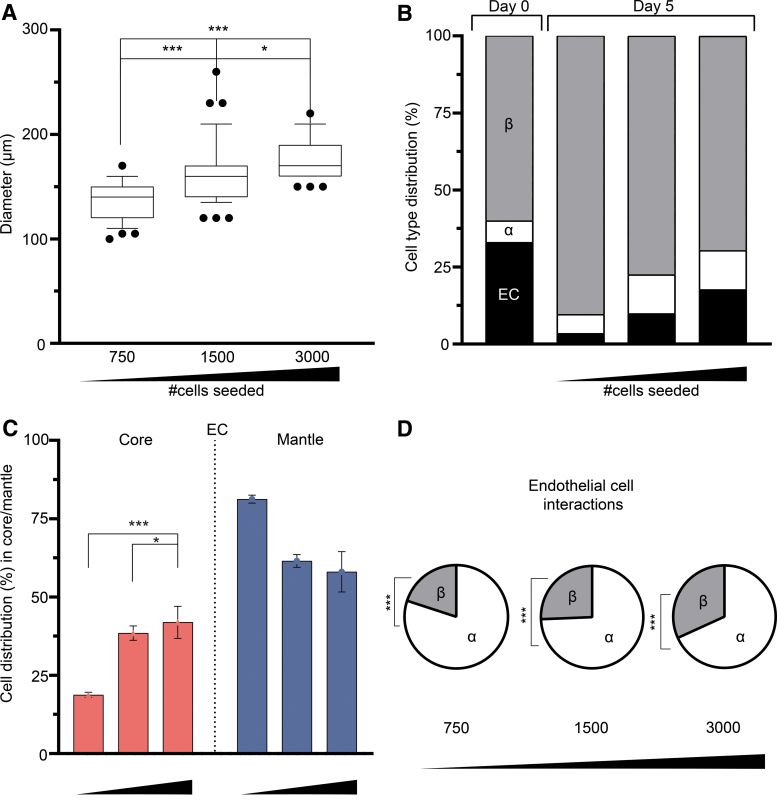
Pseudoislet size (number of cells) affects the presence of endothelial cells. **(A)** Three differently sized pseudoislets were generated, comprising a total number of 750, 1500, and 3000 cells, and the resulting mean diameters significantly increased with greater cells numbers at day 5 (*p < 0.025, ***p < 0.0001). **(B)** Day 0 represents the initial cell distribution. After 5 days in culture, the percentage of endothelial cells increased (3.5% to 8.6% to 18.5%) with the size of the pseudoislet. In contrast, the percentage of β cells decreased (90.5% to 79.7% to 69.2%) with increasing pseudoislet size. The percentage of α cells increased (6.1–11.7%) between the 1500 and 3000 cell pseudoislet, which persisted (12.3%) in the 3000 cell pseudoislet. **(C)** Endothelial cells became significantly more prevalent in the core of the pseudoislet when their numbers increased (*p < 0.025, ***p < 0.0002). **(D)** The endothelial cell affinity for α and β cells was not affected by the change in the pseudoislet size (***p < 0.004). Results are expressed as mean ± SEM or 10–90 percentile and each data set includes 13 pseudoislets (*n* = 13), and the experiment was repeated three times (*N* = 3).

As given in Figure 4A, the resulting pseudoislets observed at day 5 had an average diameter of 136.8 ± 2.9 μm, 163.2 ± 5.0 μm, and 176.4 ± 2.8 μm for 750, 1500, and 3000 cells, respectively. We then compared the relative cell populations among the differently sized pseudoislets. As the size of the pseudoislet increased, the mean percentage of β cells concomitantly decreased from 90.5% ± 2.5% to 79.7% ± 6.1% to 69.2% ± 2.8%. In contrast, endothelial cells were increasingly prevalent, from 3.5% ± 0.4% to 8.6% ± 1.2% to 18.5% ± 2.3%, as the pseudoislet size increased (Fig. 4B and Supplementary Fig. S4A). Similar to endothelial cells, the prevalence of α cells also increased as the islet size increased, although with smaller percentage increases (6.1% ± 0.4% to 11.7% ± 0.8% to 12.3% ± 0.6%) (Fig. 4B and Supplementary Fig. S4A). There was no impact on the viability of the cells between the three different sized pseudoislets at day 10 (Supplementary Fig. S5C). Taken together, our findings demonstrated that increasing the size of the pseudoislet concomitantly affected the prevalence of β and endothelial cells (*p* < 0.0001).

To determine whether pseudoislet size affected cell type localization, we quantified whether the different cell types were found in the mantle or core. We found that increasing the pseudoislet size also resulted in significantly greater numbers of endothelial cells in the core (Fig. 4C) (*p* < 0.0002). Still, the size of the pseudoislet did not affect the preferential localization of α cells to the mantle or β cells to the core (Supplementary Fig. S4B, C, *p* < 0.001). The pseudoislet size significantly affected the number of proliferating cells; as the size increased, the number of positive EdU labeled cells decreased (from 59.3% to 45.3% to 37.5% at day 10) (Supplementary Fig. S5A, B; *p* < 0.010). Regardless of the changes in the localization of the endothelial cells, their affinity toward α cells was persistent (Fig. 4D and Supplementary Fig. S4D; *p* < 0.004).

## Discussion

In this study, we were interested in understanding the rules governing the self-assembly of pseudoislets, specifically the role of α and β cells in the incorporation of endothelial cells in the pseudoislets. As the pseudoislets formed, we first observed that β cells were prevalently located in the core compared with endothelial cells, which were primarily located in the mantle. Αlpha cells were approximately evenly distributed between the mantle and core (Fig. 1G).

By altering the seeding ratios of the three cell types comprising the pseudoislets, we could determine whether the prevalence of α and β cells would affect the presence of endothelial cells. We observed that the self-assembly process is highly regulated, as the numbers of α, β, and endothelial cells converged on a specific ratio regardless of the initial seeding ratio (Fig. 3 and Supplementary Fig. S5D). Furthermore, the prevalence of endothelial cells increased, whereas that of β cells decreased when the size of the pseudoislet increased (Fig. 4B, C and Supplementary Fig. S5D). Another important finding was that we observed that the preferential proximity between α and endothelial cells remained unchanged in all tested conditions (Figs. 2A, B, 3D, and 4D).

One limitation of this study is its reliance on cell lines and not primary tissue. This choice was considered carefully in light of the aims to challenge the self-assembly of pseudoislets by using different ratios of three cell types, which would not be possible with primary cells. With this system, it was possible to control the composition of the pseudoislet, thereby leading to a better understanding of the cell-regulated interactions between α, β, and endothelial cells.

To compare our findings with those on primary cells, the ratio of cells we obtained in the pseudoislets (21% α cells, 74% β cells, and 5% endothelial cells) resembled that of a rodent islet: 19% α cells, 75% β cells, and 6% endothelial cells.^23,24^ Earlier studies have demonstrated that *in vivo*, endothelial cells comprise 1–2% of the tissue mass of the islet of Langerhans, which also contains additional cell types such as delta (δ), epsilon, and pancreatic polypeptide-producing cells.^33^ The spatial arrangement in the islets of Langerhans greatly differs between species and changes between normal physiology and a pathophysiological state as well as with islet size.^24,34,35^

This study found that β cells were prevalently located in the core compared with endothelial cells, which were primarily located in the mantle (Fig. 1G). Αlpha cells were approximately evenly distributed between the mantle and core. It has been shown that hyperglycemia for 4 weeks in a rodent model caused the relocation of α cells from the mantle to the core of the islet.^36^ These changes do not only affect the function of the islet, such as glucagon and insulin secretion, but also the overall cell distribution of α and β cells inside it.^37^

The reasons explaining why some cell types have a preferential localization in the islet and why this localization changes under certain conditions remained to be elucidated. The most interesting finding we observed was that the preferential proximity between α and endothelial cells remained unchanged in all tested conditions (Figs. 2A, B, 3D, and 4D).

In agreement with this study, an interaction between α and endothelial cells has been reported in two other settings, although not in the case of pseudoislets. First, it has been shown that α cells respond to glucose with an increased intracellular Ca^2+^ concentration,^23,38^ which then influences angiogenesis and proliferation of endothelial cells.^39,40^

Second, in the development of the human islet of Langerhans, it has been seen that endothelial cells have an increased preference to neighbor α cells. At 19–21 weeks of gestational age, two separate cell clusters, one composed of β cells and another composed of α and δ cells, fuse to create the islet of Langerhans. Of note, before the development of the islet of Langerhans, the endothelial cells are preferentially found in the α/δ cell cluster and are rare in the β cell cluster.^12,41^ Finally, in adult islets of Langerhans, studies have shown that most α cells are in direct contact with endothelial cells,^22,23^ although it was not known until the present study whether this affinity would hold up under an experimental challenge. The fact that α and endothelial cells have a preferential proximity indicates a potentially important role for the α cells in the incorporation of endothelial cells into the pseudoislet.

This study demonstrates that the prevalence of endothelial cells increases, whereas that of β cells decreases when the size of the pseudoislet increases (Fig. 4B, C). This increase in endothelial cells is important because they provide structure and extracellular matrix (ECM), and they have been shown to increase endocrine function both *in vivo* and *in vitro.*^7,20,21,42^

Indeed, the crosstalk between endothelial and endocrine cells can result in increased insulin release, cell proliferation, and differentiation.^10,11^ For example, the ECM comprising laminins and collagen IV produced by endothelial cells has been shown to enhance insulin secretion and β cell differentiation.^7,11^ In fact, β cells have a polarized insulin secretion toward the endothelial cells and the ECM they produced.^43^

Of interest, the loss of endothelial ECM has a detrimental effect on β cell insulin secretion.^44^ Despite the benefits of endothelial cells, it has been shown in other studies that smaller islets (40–60 μm in diameter) do not incorporate endothelial cells,^22^ and that increasing the size of the human islet of Langerhans results in a decreased number of β cells.^45^

In addition, we observed minor increases in α cell numbers as the pseudoislet increased in size. This finding is supported by other *in vitro* evidence that larger islet size increased the number of α cells present within.^22,34^ Regarding our results, it is important to note that the endothelial cells inside the pseudoislet do not form a vessel structure. Instead, the endothelial cells are there to support the pseudoislet, and have been shown in multiple studies to have a positive effect on the pseudoislet function upon transplantation.^7,10,11,20,21,42^ For example, HUVECs combined with human adipose-derived stem cells had positive effects on human induced pluripotent stem cell-derived endocrine progenitors to generate multicellular spheroids that are comparable with human islets both in their maintenance of glucose homeostasis and in size.^2^

Taken together, our work suggests that α cells play an important role to the endothelial cells. This is a relevant finding for the tissue engineering field, in which future strategies might rely on newly generated cells to create bioengineered islets to treat patients.

## Supplementary Material

Supplemental data

Supplemental data

Supplemental data

Supplemental data

Supplemental data

Supplemental data
