# Supplementary material for: The Role of Alpha Cells in the Self-Assembly of Bioengineered Islets
Source: Tissue Eng Part A. 2021 Aug 16;27(15-16):1055–63. doi: 10.1089/ten.tea.2020.0080 (PMC8392094; doi:10.1089/ten.tea.2020.0080)
Supplement: Supplemental data [file Supp_Table1.docx]

Supplementary Table 1. All primary and secondary antibodies and dye.

| Antigen | Host species | Dilution | Source | RRID |
| --- | --- | --- | --- | --- |
| Primary antibodies |  |  |  |  |
| CD31/PECAM-1 | Goat (polyclonal) | 1/20 (10 µg/mL) | R&D systems | (R and D Systems Cat# AF3628, RRID:AB_2161028) |
| Insulin | Rabbit (polyclonal) | 1/500 (4 µg/mL) | Abcam | (Abcam Cat# ab63820, RRID:AB_1925116) |
| Glucagon | Mouse (monoclonal K79bB10) | 1/2350 (4 µg/mL) | Sigma-Aldrich | (Sigma-Aldrich Cat# G2654, RRID:AB_259852) |
| Secondary antibodies |  |  |  |  |
| Goat IgG Alexa Fluor 647 nm | Donkey (polyclonal) | 1/500 (4 µg/mL) | Invitrogen |  |
| Rabbit IgG Alexa Fluor 568 nm | Goat (polyclonal) | 1/500 (4 µg/mL) | Invitrogen |  |
| Mouse IgG Alexa Fluor 488 nm | Goat (polyclonal) | 1/500 (4 µg/mL) | Invitrogen |  |
| Dye |  |  |  |  |
| DAPI |  | 0.7 µg/mL | Sigma-Aldrich |  |
