# Supplementary material for: The Role of Alpha Cells in the Self-Assembly of Bioengineered Islets
Source: Tissue Eng Part A. 2021 Aug 16;27(15-16):1055–63. doi: 10.1089/ten.tea.2020.0080 (PMC8392094; doi:10.1089/ten.tea.2020.0080)

Supplementary Figure 1. (A) The pseudoislets were imaged to a depth of 80 µm. (B) The .nd2 files were processed with Fiji^25^ to identify single cells per z-stack. The cell counter plugin was used to count every cell type to a depth of 50 µm into the pseudoislet (beginning the quantification after the first 10 µm). Double counts were diminished by going back and forth between z-stacks to remove duplicates. (C) All quantifications were exported into Excel (Microsoft). The data were normalized using a convex combination to correct for the different cell numbers per cell type. The normalized data were exported into Prims 8.2 (GraphPad) for statistical analysis and graph making.


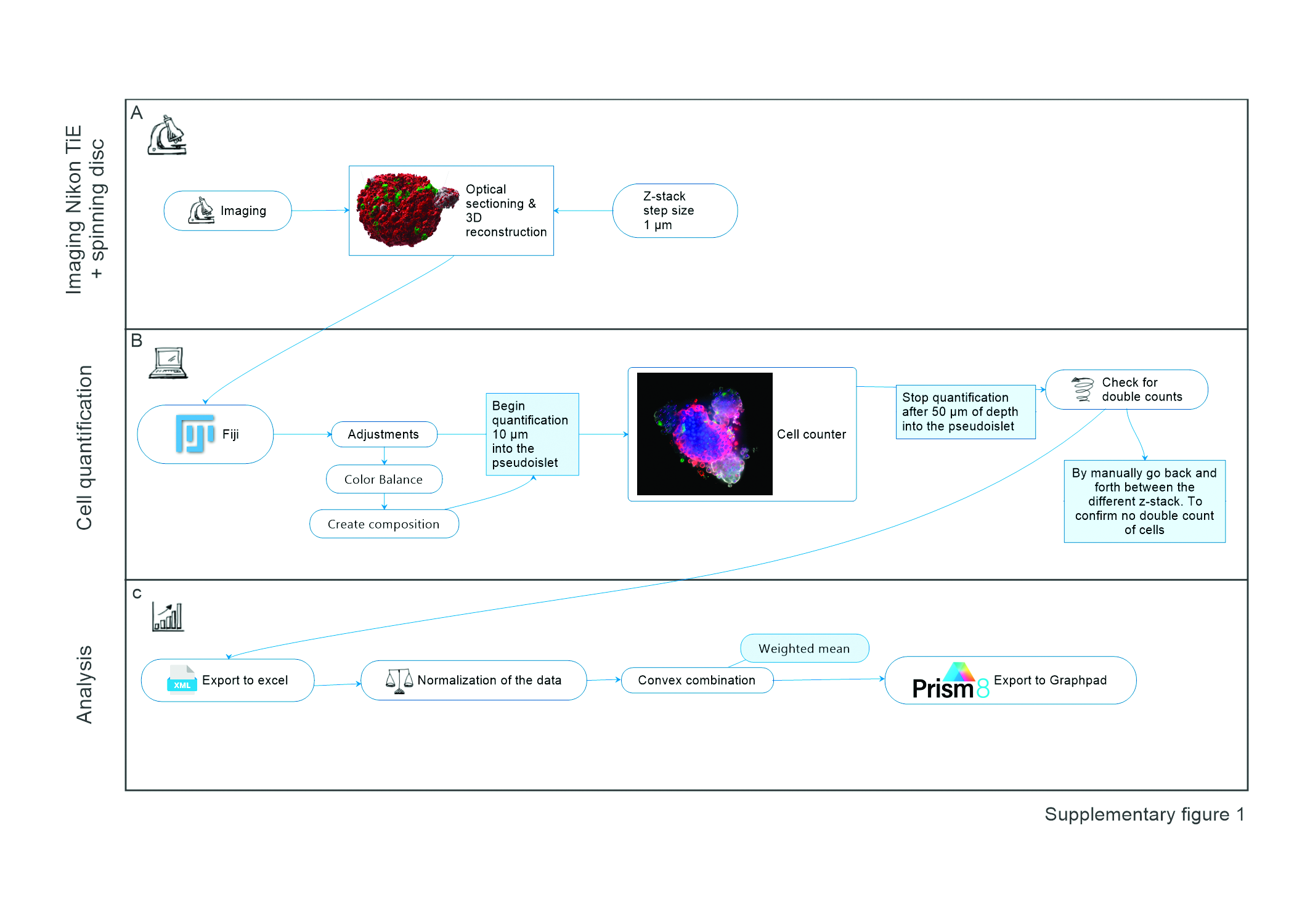

Supplement: Supplemental data [file Suppl_FigureS1.docx]
