# Supplementary material for: The Role of Alpha Cells in the Self-Assembly of Bioengineered Islets
Source: Tissue Eng Part A. 2021 Aug 16;27(15-16):1055–63. doi: 10.1089/ten.tea.2020.0080 (PMC8392094; doi:10.1089/ten.tea.2020.0080)
Supplement: Supplemental data [file Suppl_FigureS2.docx]

Supplementary Figure 2. (A) The pseudoislets were analysed of a total depth of 50 µm. First, every cell type was classified and tracked by the Fiji plugin “cell counter”. To ensure no double counts, it was necessary to go back and forth between adjacent z-stacks. (B) One example of classifying the interactions of an α cell inside a pseudoislet. To quantify the interactions, one α cell was marked with (α*) and every surrounding cell type was classified. In this example, one α cell interacts with two β cells and one endothelial cell. However, pseudoislets are 3D spheroids and it is therefore necessary to consider cell interactions in the z-axis. This is demonstrated in the next image that shows the difference from 20 to 25 µm in depth of the pseudoislet. Here we see that the same α cell also had two more interactions: one β cell and one endothelial cell. (C) One example of classifying the interactions of a β cell inside a pseudoislet. To quantify the interactions, one β cell was marked with (β*) and every surrounding cell type was classified. In this example, one β has an interaction with two α cells and one β cell. The arrow indicates that the distance between the β* and endothelial cell is too far to be classified as an interaction. Viewing the adjacent z-image resulted in two additional interactions being attributed to this cell: one α cell and one β cell.
