# Supplementary material for: The Role of Alpha Cells in the Self-Assembly of Bioengineered Islets
Source: Tissue Eng Part A. 2021 Aug 16;27(15-16):1055–63. doi: 10.1089/ten.tea.2020.0080 (PMC8392094; doi:10.1089/ten.tea.2020.0080)
Supplement: Supplemental data [file Suppl_FigureS5.docx]

Supplementary Figure 5. (A) The percentage EdU positive cells of each size of pseudoislet at day 5 and 10. The larger (3000 cells) pseudoislets have a lower number of proliferating cells compare to the smaller (750 cells) pseudoislets (**P* < 0.014, ****P* < 0.004). (B) Immunohistochemistry staining showing the proliferating positive cells (EdU; red) and nuclei (DAPI; blue) at day 10. (C) Viability staining showing dead cells (red) and nuclei (green) at day 10. (D) Immunohistochemistry staining showing nuclei (DAPI; blue), α cells (glucagon; green), β cells (insulin; red) and endothelial cells (CD31; grey) in all conditions. Scale bars: 50 µm. Results are expressed as mean ± SD and each data set includes 12 pseudoislets (n = 12), and the experiment was repeated one time (N = 1)
