# Supplementary material for: The Role of Alpha Cells in the Self-Assembly of Bioengineered Islets
Source: Tissue Eng Part A. 2021 Aug 16;27(15-16):1055–63. doi: 10.1089/ten.tea.2020.0080 (PMC8392094; doi:10.1089/ten.tea.2020.0080)
Supplement: Supplemental data [file Suppl_FigureS3.docx]

Supplementary Figure 3. (A) After five and ten days in culture, the number of both α and endothelial cells deviated from the seeding quantity, with α cells increasing and endothelial cells decreasing over time (****P* < 0.001). (B) The cell type distribution for each pseudoislet measures is shown. In contrast, α cells statistically significantly increased and endothelial cells decreased between day 5 and 10 (**P* < 0.05, ****P* < 0.001). (C) The core and mantle distribution of each cell type between day 5 and 10 (**P* < 0.05, ****P* < 0.001). (D) The preferential proximity between the three cell types in the different seeding conditions resulted in identical preferential proximity after 10 days in culture. Endothelial cells had preferential proximity with α cells in all conditions (****P* < 0.001), and β cells always had a preferential affinity for α cells (****P* < 0.001). The α cells had preferential proximity to β over endothelial cells in two conditions (**P* < 0.05). Results are expressed as mean ± SEM or 10 to 90 percentile and each data set includes 13 pseudoislets (n = 13), and the experiment was repeated three times (N = 3)
