# Supplementary material for: The Role of Alpha Cells in the Self-Assembly of Bioengineered Islets
Source: Tissue Eng Part A. 2021 Aug 16;27(15-16):1055–63. doi: 10.1089/ten.tea.2020.0080 (PMC8392094; doi:10.1089/ten.tea.2020.0080)
Supplement: Supplemental data [file Suppl_FigureS4.docx]

Supplementary Figure 4. (A) The cell count of each cell type in the various pseudoislet sizes (***P* < 0.002, ****P* < 0.001). (B) Total core and mantle cell distribution of α cells in the variously sized pseudoislets (****P* < 0.004). (C) Total core and mantle cell distribution of β cells in the variously sized pseudoislets (****P* < 0.004). (D) The preferential proximity between the three cell types in the differently sized pseudoislets. **Endothelial cells had preferential proximity to be in contact with α cells in all conditions (****P* < 0.004).** β cells had a preferential affinity for α cells, which did not change between the different seeding conditions (****P* < 0.001). The α cells had equal preferential proximity to both β and endothelial cells except in the 3000 cell pseudoislet, where they were found in proximity to β cells (****P* < 0.001). Results are expressed as mean ± SEM or 10 to 90 percentile and each data set includes 13 pseudoislets (n = 13), and the experiment was repeated three times (N = 3)
